# COMP-angiopoietin-1 mitigates changes in lipid droplet size, macrophage infiltration of adipose tissue and renal inflammation in streptozotocin-induced diabetic mice

**DOI:** 10.18632/oncotarget.21998

**Published:** 2017-10-23

**Authors:** Yu Jin Jung, Woong Park, Tung Nguyen-Thanh, Kyung Pyo Kang, Heung Young Jin, Suhn Hee Kim, Wonhee Suh, Won Kim

**Affiliations:** ^1^ Department of Internal Medicine, Division of Nephrology, Chonbuk National University Medical School, Jeonju, Republic of Korea; ^2^ Department of Internal Medicine, Division of Endocrinology, Chonbuk National University Medical School, Jeonju, Republic of Korea; ^3^ Research Institute of Clinical Medicine of Chonbuk National University-Chonbuk National University Hospital, Jeonju, Republic of Korea; ^4^ Department of Physiology, Chonbuk National University Medical School, Jeonju, Republic of Korea; ^5^ College of Pharmacy, Chung-Ang University, Seoul, Republic of Korea

**Keywords:** angiopoietin-1, adipose tissue, fat droplet, kidney, inflammation

## Abstract

Adipose tissue is considered to be an endocrine organ, and adipocyte size correlates with insulin resistance and metabolic parameters in obesity. There is little data on the effects of angiopoietin-1 in adipose tissue and kidney in streptozotocin (STZ)-induced diabetes. In this study, we investigated the protective effect of COMP-angiopoietin-1 (COMP-Ang1), a potent variant of angiopoietin-1, on vascular endothelial cells in epididymal adipose tissue and its regulatory effect on other metabolic parameters, such as lipid droplet diameter, macrophage infiltration, and renal inflammation in STZ-treated mice. Our data showed that COMP-Ang1 increased the density of platelet endothelial cell adhesion molecule-1 (PECAM-1)-1-positive vascular endothelial cells in adipose tissue, which were significantly decreased by treatment with STZ. COMP-Ang1 ameliorated the STZ–induced decrease in lipid droplet diameter and increase in macrophage infiltration in adipose tissue. Serum free fatty acid and triglyceride levels were decreased after administration of COMP-Ang1. There was a beneficial effect on serum insulin levels after treatment with COMP-Ang1 in STZ-induced diabetic mice. Fasting blood glucose levels in COMP-Ang1-treated mice were significantly lower than those of LacZ-treated mice. Cotreatment with COMP-Ang1 and STZ also had similar effects on the above parameters. Administration of soluble Tie2, an inhibitor of angiopoietin-1, reversed the effects of COMP-Ang1. COMP-Ang1 was found to ameliorate the up-regulation of proinflammatory molecules and F4/80-positive macrophage infiltration in the kidneys of STZ-treated mice. COMP-Ang1 increased the phosphorylation of Akt in epididymal adipose tissue and kidneys of STZ-induced diabetic mice. These data indicate that COMP-Ang1 regulates lipogenic effects in adipose tissue and renal inflammation in STZ-induced diabetic mice.

## INTRODUCTION

Adipose tissue is regarded as an endocrine organ that produces cytokines and adipokines [1]. Adipose tissue is composed of adipocytes, preadipocytes, vascular endothelial cells, fibroblasts, and macrophages [2]. Adipocytes play important roles in both normal and diabetic metabolic regulation. Several studies have shown that adipocyte size is related to adipocyte insulin sensitivity and adipokine secretion [3–5]. Omental adipocyte size has been shown to correlate with insulin resistance and metabolic parameters in obese individuals [6]. It has been suggested that inflammatory cytokines in adipose tissues are associated with insulin resistance [7]. Therefore, increased adipocyte size in obesity is linked to obesity-related metabolic disorders. However, the size of adipocytes in type 1 diabetes is different from that in obesity-related metabolic disorders. Streptozotocin (STZ) decreases fat mass and also suppresses fat cell diameter, an index of fat metabolism [8]. Szkudelska et al. [9] demonstrated induction of adipocyte dysfunction in diabetic rats by STZ and nicotinamide. They suggested that an imbalance between lipogenesis and lipolysis in adipose tissue reduces lipid accumulation in STZ- and nicotinamide-induced diabetes. STZ also has a direct effect on lipolysis in adipocytes [10]. Thus, down-regulation of STZ-induced lipolytic effects may ameliorate metabolic parameters.

Adipocyte cytokines are regulated by adipose tissue macrophages [11]. Fatty acids and adipokines are released directly into the portal circulation from adipose tissue. STZ-induced insulin deficiency increases serum plasma triglyceride levels in mice and decreases lipolysis [12]. As adipose tissue macrophages have critical functions in metabolic disorders, regulation of adipose tissue macrophages may provide a new avenue in treatment of metabolic disorders.

STZ has been used to induce diabetic nephropathy characterized by albuminuria and glomerular hypertrophy. Dysfunction and inflammation of endothelial cells in the kidney are critical factors related to diabetic nephropathy [13]. It has been demonstrated that high glucose levels can increase proinflammatory molecules such as intercellular adhesion molecule (ICAM)-1 and vascular cell adhesion molecule (VCAM)-1 in endothelial cells [14]. Recently, vascular endothelial growth factor-A inhibitor ameliorated renal inflammation in a mouse model of type 1 diabetes [15]. Thus, down-regulation of renal endothelial activation, especially in the early stage, may have a protective role against diabetic nephropathy. However, there are not many reports about the early anti-inflammatory effects on diabetic nephropathy.

Angiopoietin-1 (Ang1), a potent angiogenic factor, acts on the Tie-2 receptor tyrosine kinase, and is linked to vascular endothelial cell survival, morphogenesis, and capillary permeability [16, 17]. Ang1 maintains endothelial survival in the presence of potentially lethal stimuli through phosphorylation of the serine-threonine kinase, Akt [18]. COMP-Ang1, a variant of native Ang1 engineered by replacing the N-terminus of Ang1 with cartilage oligomeric matrix protein (COMP), has been shown to be more potent than native Ang1 with respect to the ability to phosphorylate Tie2 in endothelial cells [19]. It has been demonstrated that adipose tissue growth is regulated by Ang1 [20]. Recently, we demonstrated that COMP-Ang1 ameliorated perturbations of the diameter of epididymal adipocyte fat droplets and of metabolic parameters in a type 2 diabetes model [21]. However, there is little data on the effects of Ang1 on lipid droplet sizes, adipocyte macrophage infiltration, metabolic parameters or renal inflammation in a STZ-induced diabetes model.

In the present study, we report the effects of exogenous COMP-Ang1 on vascular endothelial cells in adipose tissue, and its effects on adipocyte lipid diameter, macrophage infiltration, and serum free fatty acid (FFA), serum triglycerides, serum insulin, glucose levels, ICAM-1, VCAM-1 expression and macrophage recruitment in the kidney with a mouse model of diabetes induced by STZ.

## RESULTS

### COMP-Ang1 ameliorates STZ-induced decrease of PECAM-1-positive vascular endothelial cell density in epididymal adipose tissue

**Figure 1:**
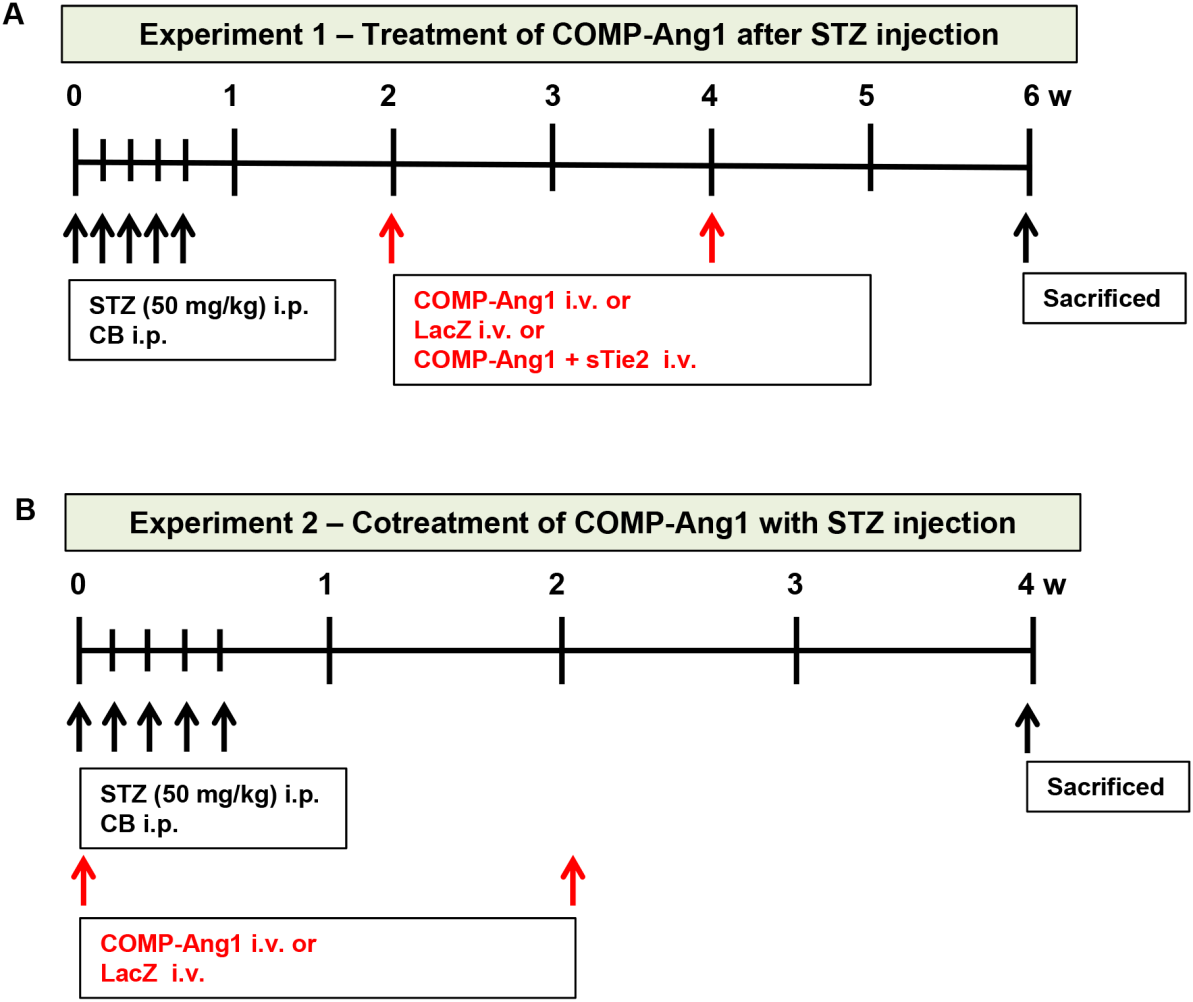
Schematic diagram of experiments 1 and 2 **(A)** To evaluate the therapeutic effects of COMP-Ang1, we injected COMP-Ang1 adenovirus 2 w after streptozotocin (STZ) or control buffer (CB) administration and harvested adipose tissue, and blood samples 4 w after COMP-Ang1 adenovirus treatment. **(B)** To evaluate the preventive effects of COMP-Ang1, we coadministered COMP-Ang1 adenovirus simultaneously with STZ and harvested adipose tissue, and blood samples 4 w after COMP-Ang1 adenovirus treatment.

**Figure 2:**
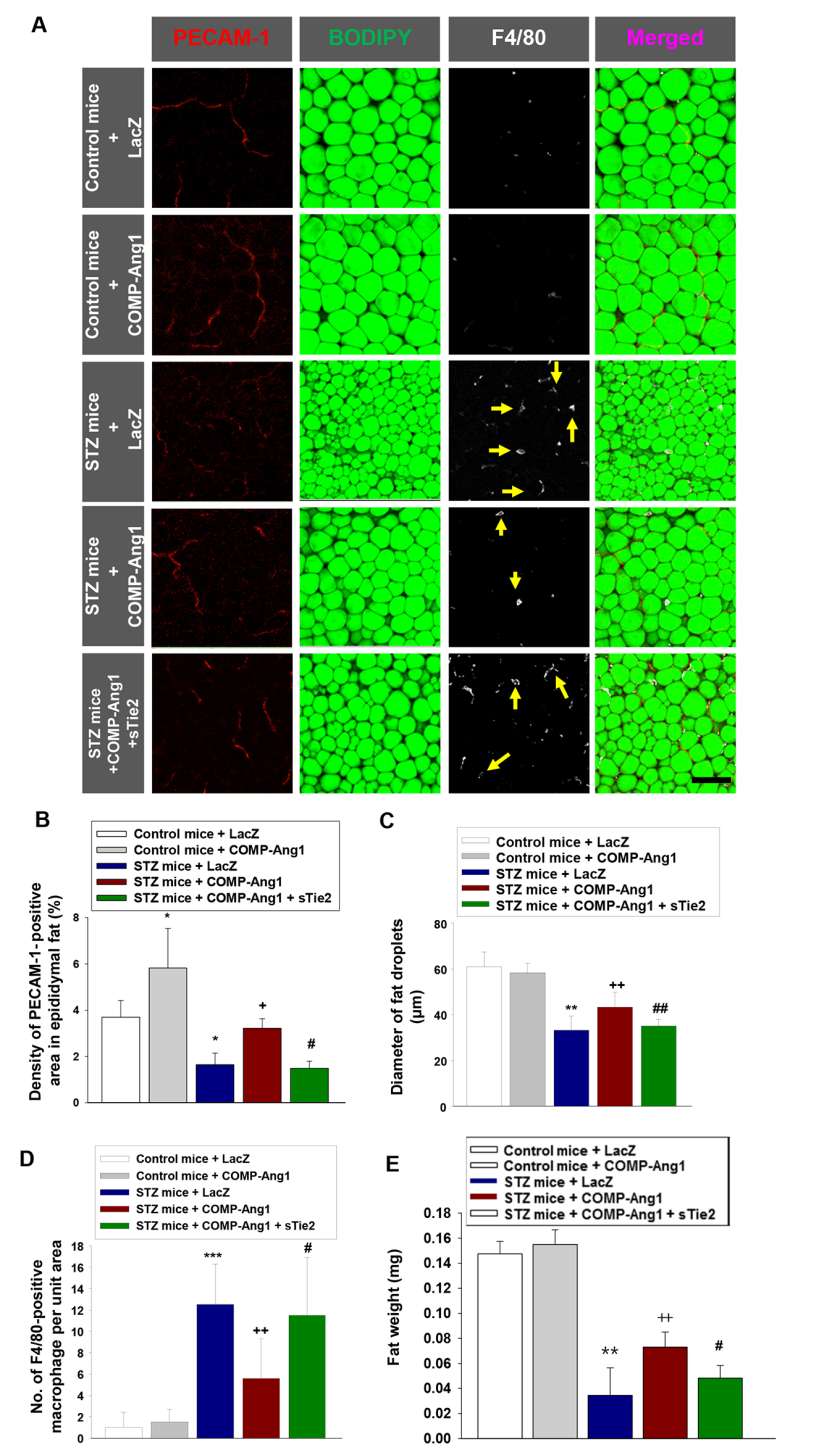
Changes of vascular endothelial cells, fat droplets and macrophage infiltration in adipose tissue **(A-D)** Representative images of platelet endothelial cell adhesion molecule-1 (PECAM-1), BODIPY, and F4/80 staining of epididymal adipocytes. Adipose tissue from mice that received control buffer (Control mice) plus LacZ, Control mice plus COMP-Ang1 adenovirus, streptozotocin (STZ mice) plus LacZ, STZ mice plus COMP-Ang1 adenovirus, or STZ plus COMP-Ang1 adenovirus plus sTie2-Fc adenovirus (sTie2) were harvested 4 w after injection with LacZ or COMP-Ang1 adenovirus. Mice were pretreated with 1×10^9^ PFU Ade-sTie2 24 h before treatment with 1 x 10^9^ PFU Ade-COMP-Ang1. Adipose tissue was whole mounted for immunofluorescence staining. (A) Fat droplets in endothelial cells were visualized by PECAM-1 (red) and BODIPY immunofluorescence staining (green), and macrophages by F4/80 (white), and the images were merged. Yellow arrows indicate infiltration of F4/80 positive macrophages. Scale bar = 100 μm. (B-D) Quantification of PECAM-1-positive endothelial cells (B), epididymal adipocyte fat droplet diameters (C), and F4/80-positive macrophage infiltration (D) in epididymal adipose tissue. **(E)** Weight of adipose tissue. Adipose tissue was harvested as described above. The values are mean ± SD for six animals in each group. ^*^P<0.05 vs. Control mice+LacZ; ^**^P<0.01 vs. Control mice+LacZ; ^***^P<0.001 vs. Control mice+LacZ; + *p*<0.05 vs. STZ mice+LacZ; ++ *p*<0.01 vs. STZ mice+LacZ; # *p*<0.05 vs. STZ mice+COMP-Ang1; ## *p*<0.01 vs. STZ mice+COMP-Ang1.

To evaluate the effect of COMP-Ang1 on vascular endothelial cells in epididymal adipose tissue, we administered COMP-Ang1 adenovirus 2 w after STZ injection (Figure 1, experiment 1). The density of PECAM-1-positive endothelial cells in epididymal adipose tissue was evaluated by whole mount staining. The PECAM-1-positive density in epididymal adipose tissue of STZ-induced diabetic mice was significantly lower than that of mice treated with control buffer. COMP-Ang1 significantly ameliorated the STZ-induced decrease in the density of PECAM-1-positive cells in epididymal adipose tissue of STZ-treated mice. sTie2 adenovirus treatment reversed the effect of COMP-Ang1 on PECAM-1-positive density (Figures 2A and 2B).

### Fat droplet diameter in epididymal adipose tissue is increased by COMP-Ang1

We measured changes in adipocyte size in diabetic mice after treatment with COMP-Ang1 adenovirus. The average diameter of epididymal adipocytes in diabetic mice treated with STZ was significantly decreased, by about 55% compared to that of control buffer-treated mice (Figures 2A and 2C). Treatment with COMP-Ang1 adenovirus significantly increased the diameter of fat droplets in epididymal adipocytes of STZ-induced diabetic mice by 1.42-fold. To examine whether Tie2 affected the COMP-Ang1-induced increase of fat droplet diameters, mice were treated with sTie2 adenovirus prior to COMP-Ang1 adenovirus plus STZ treatment. Treatment with sTie2 adenovirus reversed the effect of COMP-Ang1 on the diameter of fat droplets in epididymal adipose tissue (Figures 2A and 2C).

### COMP-Ang1 mitigates the STZ-induced increase of macrophage infiltration in epididymal adipose tissue

We performed an immunofluorescence study to evaluate changes in the number of F4/80-positive macrophages in epididymal adipose tissue. Administration of STZ increased the infiltration of F4/80-positive macrophages into the adipose tissue by 12.5-fold compared to that in mice treated with control buffer. COMP-Ang1 adenovirus treatment decreased the STZ-induced accumulation of F4/80-positive macrophages by about 44.8%. Treatment with sTie2 adenovirus alleviated the COMP-Ang1-induced decrease of F4/80-positive macrophage infiltration in epididymal adipose tissue (Figures 2A and 2D). Treatment of mice with COMP-Ang1 adenovirus alone had no effect on the number of F4/80-positive macrophages (Figures 2A and 2D). STZ-induced decrease of epididymal fat weight was increased after treatment with COMP-Ang1 adenovirus (Figure 2E). To identify changes in M1 or M2 macrophages in epididymal adipose tissue, we performed quantitative real-time reverse-transcription PCR (qRT-PCR). STZ significantly increased the mRNA expression of M1 macrophage (F4/80, CD86) and M2 macrophage (CD206 [mannose receptor] and FIZZ1) markers compared with control buffer-treated mice. Treatment of diabetic mice with COMP-Ang1 adenovirus significantly diminished F4/80, CD86, CD206, and FIZZ1 mRNA expression in epididymal adipose tissue (Supplementary Figures 1A-1D). sTie2 adenovirus decreased the inhibitory effect of COMP-Ang1 adenovirus on F4/80, CD86, CD206, and FIZZ1 mRNA expression in adipose tissue (Supplementary Figures 1A-1D).

### COMP-Ang1 ameliorates STZ-induced dyslipidemia

**Figure 3:**
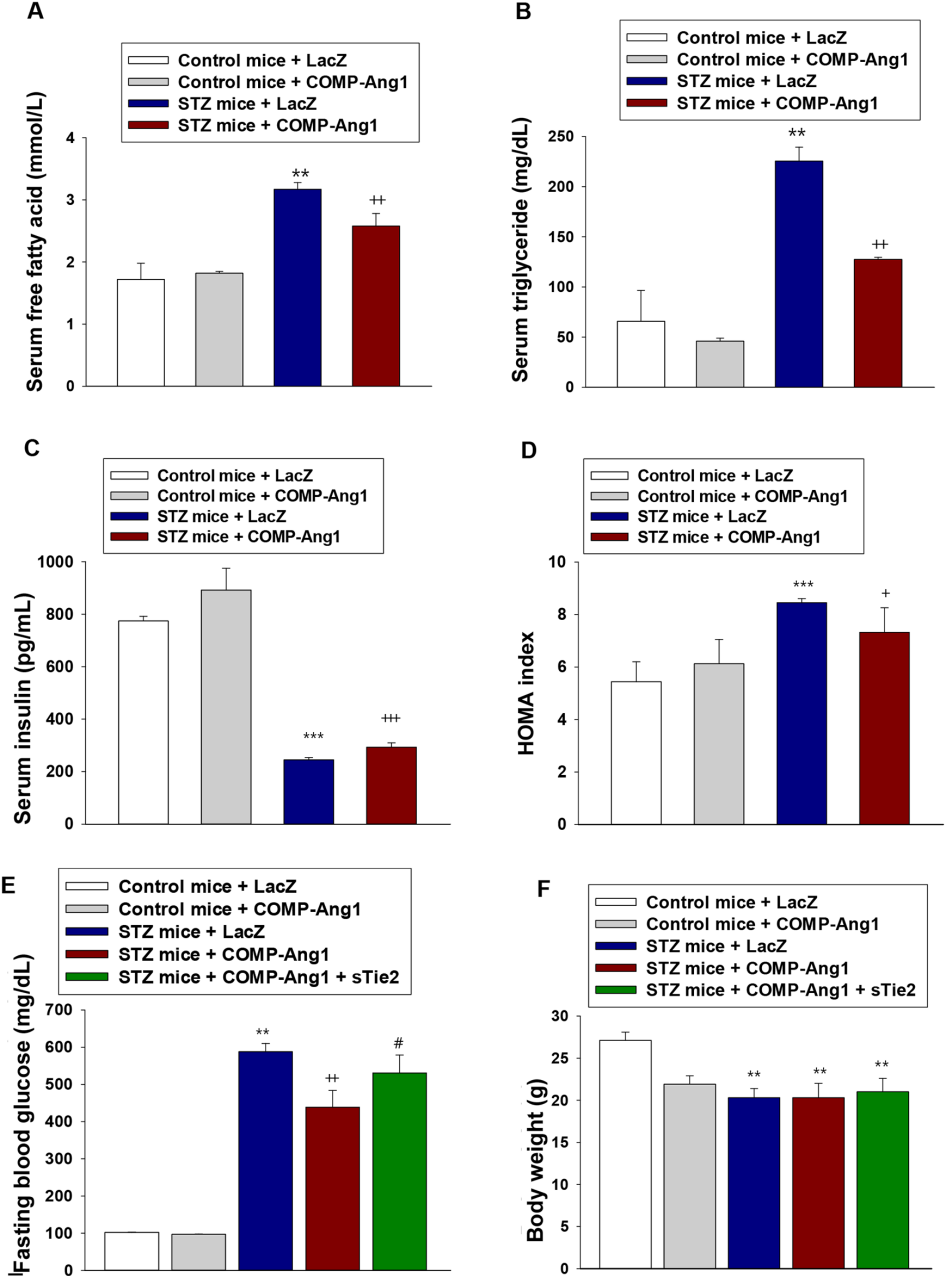
Changes of serum lipid levels and HOMA index **(A-B)** Serum levels of free fatty acids (A) and triglycerides (B). Serum levels of free fatty acids and triglycerides were measured 4 w after injection with LacZ or COMP-Ang1 adenovirus. **(C** and **D)** Serum insulin levels (C) and insulin resistance calculated by HOMA index (D). Serum insulin levels were measured by ELISA 4 w after injection with LacZ or COMP-Ang1 adenovirus. HOMA index was calculated by multiplying fasting insulin (μmol/L) by fasting glucose (mmol/L) and dividing by 22.5. **(E** and **F)** Changes in fasting blood glucose (E) and body weight (F) were measured 4 w after COMP-Ang1 adenovirus injection in control (Control mice) and STZ-induced diabetic mice (STZ mice). Data are means ± SD for six animals in each group. ^**^P<0.01 vs. Control mice+LacZ; ^***^ *P*<0.001 vs. Control mice+LacZ; + *p*<0.05 vs. STZ mice+LacZ; ++ *p*<0.01 vs. STZ mice+LacZ; +++ *p*<0.001 vs. STZ mice+LacZ; # *p*<0.05 vs. STZ mice+COMP-Ang1.

STZ injection significantly increases basal free fatty acid levels in fat cells [22]. To examine whether COMP-Ang1 reversed dyslipidemia in STZ-induced diabetic mice, we measured serum free fatty acid (FFA) and triglyceride levels. STZ treatment increased FFA and triglyceride levels compared to control buffer treatment, whereas COMP-Ang1 adenovirus administration to diabetic mice significantly reduced serum FFA and triglyceride levels (Figures 3A and 3B). COMP-Ang1 treatment alone had no effect on serum FFA or triglyceride levels.

### Serum insulin and fasting glucose levels are significantly ameliorated after treatment with COMP-Ang1 adenovirus

Concentrations of serum insulin were measured 4 w after COMP-Ang1 adenovirus injection. As shown in Figure 3C, serum insulin levels in LacZ-treated STZ mice were decreased to approximately 65% of baseline. However, a significant increase in serum insulin was observed following treatment with COMP-Ang1 adenovirus. STZ-induced increase of HOMA index was decreased after treatment with COMP-Ang1 adenovirus (Figure 3D). Blood glucose levels after COMP-Ang1 adenovirus treatment were significantly reduced compared to those after LacZ treatment (Figure 3E). No significant difference was observed between the mice treated with COMP-Ang1 adenovirus alone and LacZ alone. The body weight of the experimental animals at 4 w after COMP-Ang1 adenovirus or LacZ administration is presented in Figure 3F. Following STZ injection, all animals progressively lost body weight. COMP-Ang1 adenovirus-injected diabetic mice did not significantly change body weight (Figure 3F).

### COMP-Ang1 ameliorates mRNA levels of lipogenic genes in adipose tissue

To evaluate whether changes in lipid droplet diameter resulted from lipolysis or lipogenesis in adipose tissue, we performed qRT-PCR for genes that regulate lipolysis or lipogenesis. qRT-PCR analysis showed that COMP-Ang1 treatment ameliorated the decreased mRNA levels of lipogenic genes including *Peroxisome proliferator-activated receptor gamma (Ppar-γ)* and *CCAAT/enhancerbinding protein alpha (C/ebp-α)* in epididymal adipose tissue of STZ-treated mice (Supplementary Figures 2A and 2B). There was a tendency of increased mRNA expression of lipolytic gene including *Adipose triglyceride lipase (Atgl)* in STZ-treated mice after treatment with COMP-Ang1. However, it did not reach to the statistical significance (Supplementary Figure 2C).

### COMP-Ang1 increases Akt phosphorylation in adipose tissue of STZ-treated diabetic mice

**Figure 4:**
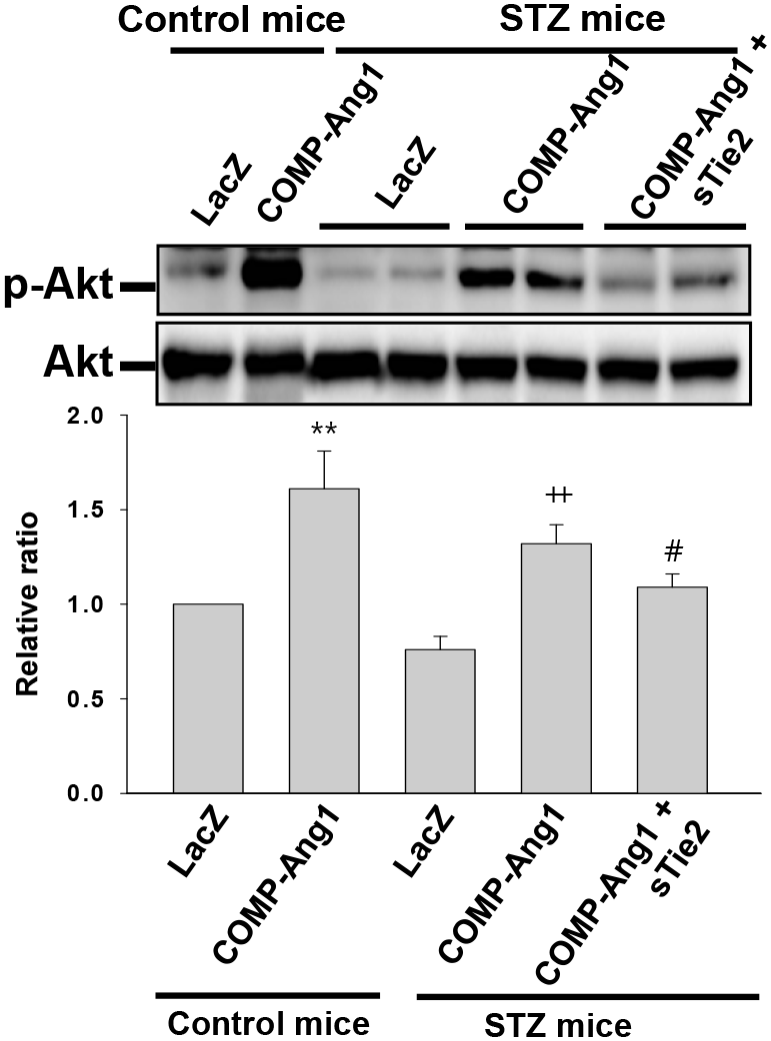
Immunoblotting analyses of phospho-Akt from adipose tissue of STZ-induced diabetic mice Protein levels of phospho-Akt and Akt in epididymal adipose tissue isolated from mice that received control buffer (Control mice) plus LacZ, Control mice plus COMP-Ang1 adenovirus, streptozotocin (STZ mice) plus LacZ, STZ mice plus COMP-Ang1 adenovirus, and STZ mice plus COMP-Ang1 adenovirus plus sTie2-Fc adenovirus (sTie2). Mice were pretreated with 1×10^9^ PFU Ade-sTie2 24 h before treatment with 1 x 10^9^ PFU Ade-COMP-Ang1. Homogenates were processed by Western blotting. Data are means ± SD. Results were similar in three independent experiments. ^**^P<0.01 vs. Control mice+LacZ ; ++ *p*<0.01 vs. STZ mice+LacZ ; # *p*<0.05 vs. STZ mice+COMP-Ang1.

Akt is a downstream mediator of Angiopoietin/Tie2 signaling. We next examined whether COMP-Ang1 regulated phosphorylation of Akt in epididymal adipose tissue of STZ-treated diabetic mice. COMP-Ang1 increased Akt phosphorylation in adipose tissue after STZ injection, compared with that in mice treated with control buffer and STZ (Figure 4). Although injection of STZ alone did not significantly activate phosphorylation of Akt in adipose tissue, treatment with COMP-Ang1 adenovirus by itself increased phosphorylation of Akt. To determine whether Tie2 is involved in COMP-Ang1-induced Akt phosphorylation in STZ-treated adipose tissue after STZ treatment, mice were administered sTie2 adenovirus before treatment with COMP-Ang1 adenovirus plus STZ. sTie2 adenovirus treatment prevented COMP-Ang1-induced Akt phosphorylaton. Therefore, COMP-Ang1-induced Akt phosphorylation occurs through Tie2 in adipose tissue (Figure 4).

### Cotreatment with COMP-Ang1 and STZ ameliorates the alterations in fat droplet diameter, vascular endothelial cell density, and macrophage infiltration observed in epididymal adipose tissue in diabetic mice

**Figure 5:**
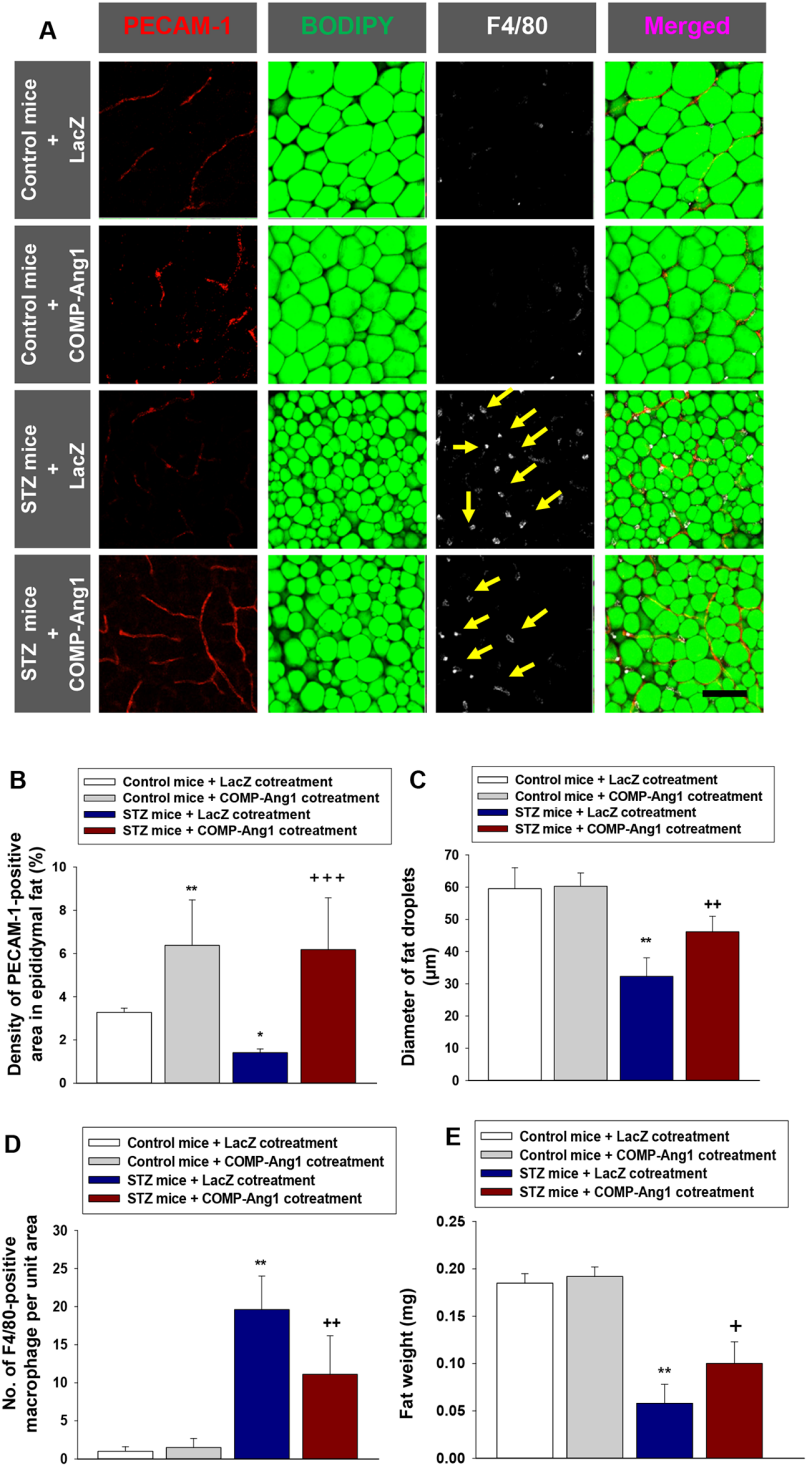
Changes of vascular endothelial cells, fat droplets and macrophage infiltration in adipose tissue **(A)** Representative images of platelet endothelial cell adhesion molecule-1 (PECAM-1), BODIPY, and F4/80 staining of epididymal adipocytes. To evaluate the preventive effect of COMP-Ang1, COMP-Ang1 adenovirus was injected at the same time with control buffer (Control mice) or STZ (STZ mice). Adipose tissue from mice that received control buffer plus LacZ (Control mice + LacZ cotreatment), control buffer plus COMP-Ang1 adenovirus (Control mice + COMP-Ang1 cotreatment), STZ plus LacZ (STZ mice + LacZ cotreatment), or STZ plus COMP-Ang1 adenovirus (STZ mice + COMP-Ang1 cotreatment) were harvested 4 w after injection with LacZ or COMP-Ang1 adenovirus. Adipose tissue was whole mounted for immunofluorescence staining. Fat droplets were visualized in endothelial cells by PECAM-1 (red) and BODIPY immunofluorescence staining (green), and macrophages by F4/80 (white), and the images were merged. Yellow arrows indicate infiltration of F4/80 positive macrophages. Scale bar = 100 μm. **(B-D)** Quantification of PECAM-1-positive endothelial cells (B), epididymal adipocyte fat droplet diameters (C), and F4/80-positive macrophage infiltration (D) in epididymal adipose tissue. **(E)** Weight of adipose tissue. The values are the means ± SD for four animals in each group. ^*^ *P*<0.05 vs. Control mice+LacZ cotreatment; ^**^ *P*<0.01 vs. Control mice+LacZ cotreatment; + *p*<0.05 vs. STZ mice+LacZ cotreatment; ++ *p*<0.01 vs. STZ mice+LacZ cotreatment; +++ *p*<0.001 vs. STZ mice+LacZ cotreatment.

To evaluate the preventive effects of COMP-Ang1 in vascular endothelial cells and on fat droplet diameters in epididymal adipose tissue, we administered COMP-Ang1 adenovirus and STZ at the same time to diabetic mice (Figure 1, experiment 2). Coadministration of COMP-Ang1 adenovirus with STZ significantly increased the density of PECAM-1-positive vascular endothelial cells and the diameters of fat droplets in epididymal adipose tissue (Figures 5A-5C). Cotreatment with COMP-Ang1 adenovirus and STZ decreased the STZ-induced accumulation of F4/80-positive macrophages by about 43% (Figures 5A and 5D). STZ-induced decrease of epididymal fat weight was increased after cotreatment with COMP-Ang1 adenovirus (Figure 5E). Cotreatment of diabetic mice with COMP-Ang1 adenovirus also significantly suppressed F4/80, CD86, CD206, and FIZZ1 mRNA expression in epididymal adipose tissue (Supplementary Figures 3A-3D).

### Cotreatment with COMP-Ang1 adenovirus and STZ also improves dyslipidemia and serum glucose levels in diabetic mice

Serum FFA and triglyceride levels after cotreatment with COMP-Ang1 adenovirus and STZ were significantly lower than those following treatment with STZ alone (Supplementary Figures 4A and 4B). Blood glucose levels 4 w after coadministration of COMP-Ang1 adenovirus and STZ were significantly reduced compared with those after treatment with STZ alone (Supplementary Figure 4C). No significant difference was observed between mice treated with COMP-Ang1 adenovirus alone and LacZ alone.

### COMP-Ang1 reduces STZ-induced renal inflammation

**Figure 6:**
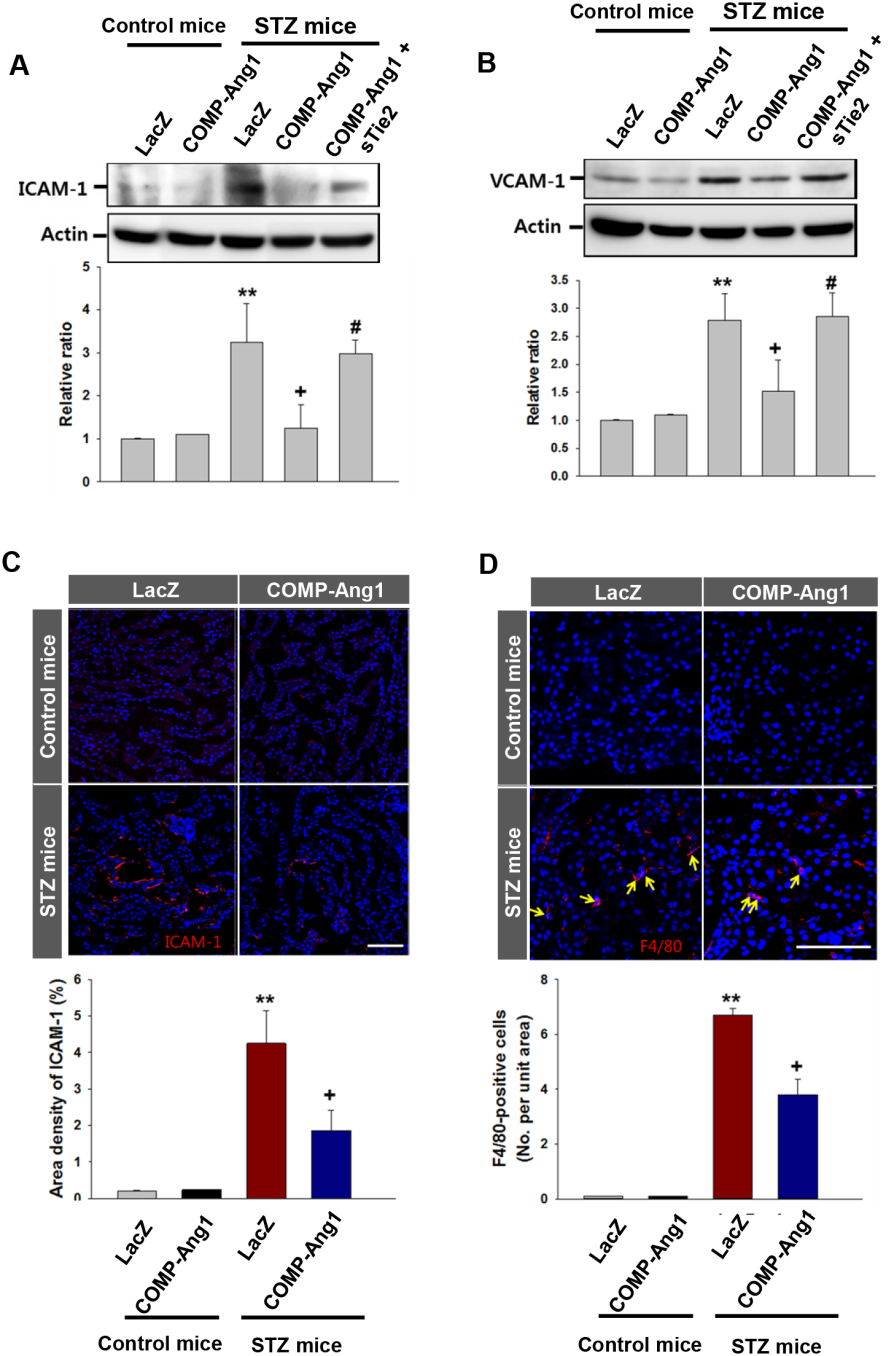
Expression of ICAM-1, VCAM-1 and F4/80 in the kidney **(A-B)** Immunoblotting analyses of intercellular adhesion molecule-1 (ICAM-1; A) and vascular cell adhesion molecule-1 (VCAM-1; B) in kidney. Mice were pretreated with 1×10^9^ PFU Ade-sTie2 24 h before treatment with 1 x 10^9^ PFU Ade-COMP-Ang1. Kidneys were harvested 6 w after control buffer (Control mice) or STZ (STZ mice) injection. Blots were probed with an anti-ICAM-1 or anti-VCAM-1 antibody. The membrane was stripped and reprobed with an anti-Actin antibody to control for protein loading in each lane. Results were similar from three independent experiments. Densitometric analyses are presented as the relative ratio of ICAM-1 or VCAM-1 to Actin. The relative ratio measured in kidneys of mice treated with control buffer plus LacZ (Control mice+LacZ) is arbitrarily presented as 1. **(C-D)** Immunofluorescence study of ICAM-1 and F4/80-positive macrophages in kidney. Kidneys were harvested at 6 w after STZ or CB injection. Tissues were fixed in 4% formaldehyde solution and kidney sections were then stained with ICAM-1 or F4/80 antibody. Scale bar = 100 μm. Lower panels shows quantitative score of ICAM-1 and F4/80 in kidney. Bar graph (C) shows the area density of the positively stained area to the total field (0.22 μm^2^). Bar graph (D) demonstrates number of F4/80-positive macrophages per high power field. Each result was similar from three independent experiments. Data are expressed as mean ± SD. ^**^, *P* < 0.01 versus Control mice+LacZ; +, *P* < 0.05 versus STZ mice+LacZ; #, *P*< 0.05 versus STZ mice+COMP-Ang1.

In the next experiment, we evaluated the inflammatory effect of STZ on the kidney and anti-inflammatory effects of COMP-Ang1 on STZ-induced renal adhesion molecule expression and macrophage infiltration. The STZ significantly increased expression of ICAM-1 and VCAM-1 in the kidney. Interestingly, COMP-Ang1 treatment resulted in a significant reduction in protein levels of ICAM-1 and VCAM-1 (Figures 6A and 6B). sTie2 reversed the effect of COMP-Ang1 on ICAM-1 and VCAM-1 expressions (Figures 6A and 6B). six weeks after STZ administration, a large amount of ICAM-1 and F4/80-positive macrophages were detected in the kidneys. However, after COMP-Ang1 treatment, the expression area of ICAM-1 and the numbers of recruited macrophages in the kidney were dramatically decreased (Figures 6C and 6D).

### COMP-Ang1 regulates phosphorylation of Akt, ERK and p65 in renal inflammation

**Figure 7:**
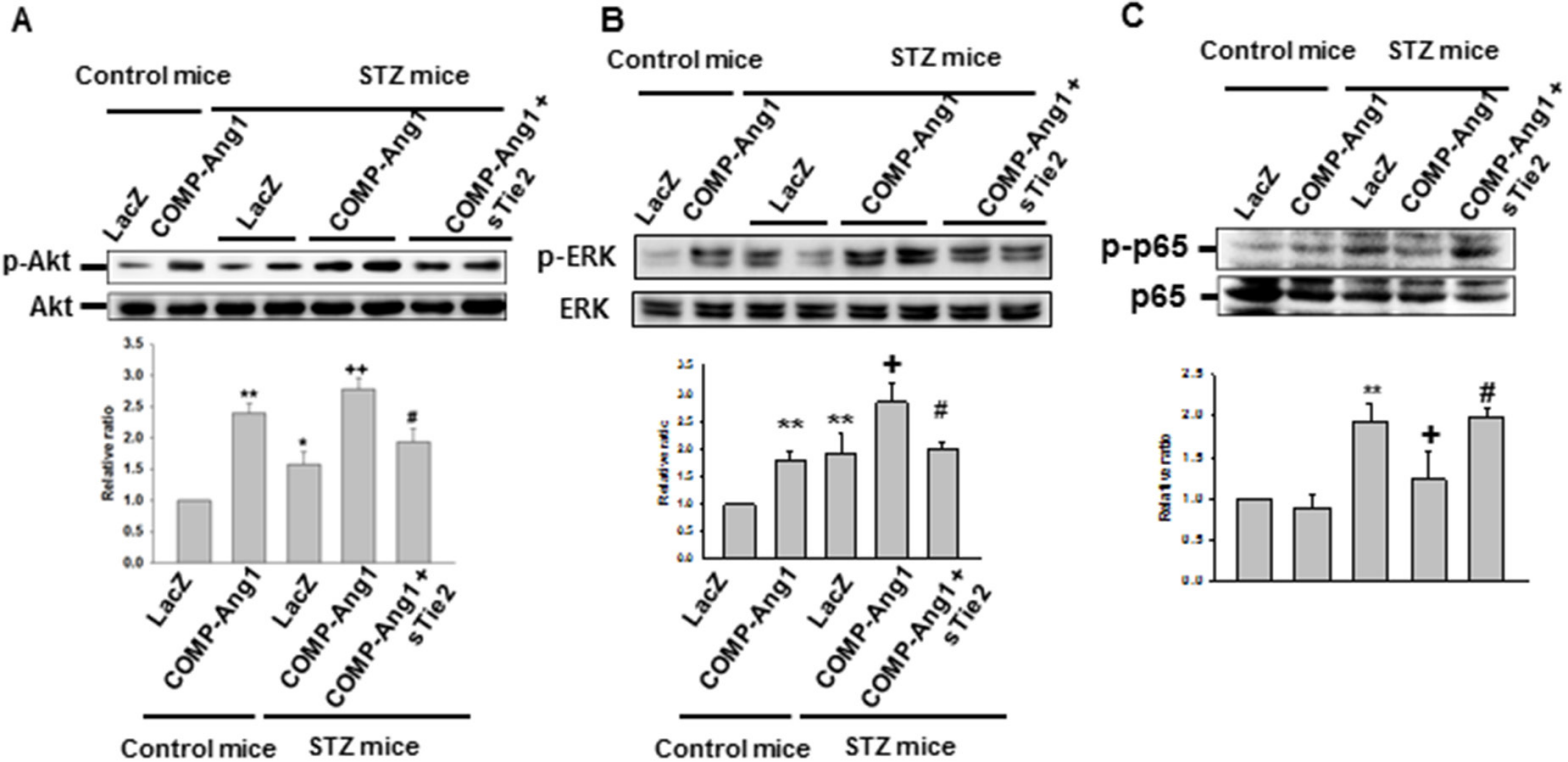
Immunoblotting analyses of phospho-Akt, phosphor-ERK and phospho-p65 from the kidney of STZ-induced mice Kidneys were harvested 6 w after STZ or control buffer (CB) injection. Mice were pretreated with 1×10^9^ PFU Ade-sTie2 24 h before treatment with 1 x 10^9^ PFU Ade-COMP-Ang1. Blots were probed with an anti-phospho-Akt (p-Akt) **(A)**, anti-phospho-ERK (p-ERK) **(B)** or phopho-p65 (p-p65) **(C)** antibody. The membrane was stripped and reprobed with an anti-Akt, anti-ERK or anti-p65 antibody to control for protein loading in each lane. Results were similar in three independent experiments. Densitometric analyses are presented as the relative ratio of p-Akt to Akt, p-ERK to ERK or p-p65 to p65. The relative ratio measured in kidneys treated with Control mice+LacZ is arbitrarily presented as 1. Data are expressed as mean ± SD. ^*^, *P*<0.05 versus Control mice+LacZ; ^**^, *P*<0.01 versus Control mice+LacZ; +, *P* < 0.05 versus STZ mice+LacZ; ++, *P* < 0.01 versus STZ mice+LacZ; #, *P*< 0.05 versus STZ mice+COMP-Ang1.

Western blot analyses showed that COMP-Ang1 significantly increased the levels of phospho-Akt in the kidney and sTie2 suppressed the effect on Akt phosphorylation of COMP-Ang1 (Figure 7A). We also found that phosphorylation of ERK was significantly increased in the kidney of COMP-Ang1-treated mice compared to LacZ-treated mice (Figure 7B). Treatment of sTie2 reversed the effect of COMP-Ang1 on ERK phosphorylation (Figure 7B). In addition, the expression of phospho-p65 in the kidney was up-regulated after treatment with STZ in the kidney, while STZ-induced phosphorylation of p65 was down-regulated after treatment with COMP-Ang1 (Figure 7C).

## DISCUSSION

In this study, we demonstrated that COMP-Ang1 had a protective effect in PECAM-1-positive vascular endothelial cells in epididymal adipose tissue in STZ-induced diabetic mice. COMP-Ang1 also ameliorated the decrease of lipid droplet diameter and increase of adipose tissue macrophage infiltration in epididymal fat tissue that were increased by STZ. Blood triglycerides, free fatty acid, insulin, and fasting glucose levels, which were increased after STZ administration, were decreased upon treatment with COMP-Ang1 adenovirus. All of our data suggest that COMP-Ang1 had a metabolic regulatory effect in diabetic mice.

Type 1 diabetes is a metabolic disorder that results in massively decreased body fat mass. As STZ is a genotoxin that induces apoptosis in pancreatic β-cells, it has been used in animal models of type 1 diabetes [23]. In addition to its toxic effect on the pancreas, STZ also has deleterious effects on adipocytes. It has been demonstrated that fat mass is markedly decreased and the mean fat cell diameter at subcutaneous, proximal epididymal, distal epididymal, perirenal and retroperitoneal fat depots are decreased in type 1 diabetes rat models [8]. In line with these findings, our data also demonstrated that STZ decreased the diameter of lipid droplets in epididymal adipose tissue.

There are two major populations of macrophages. First, there are the classically activated macrophages (M1), which secrete proinflammatory cytokines and are involved in the Th1 response. Second, there are alternatively activated macrophages (M2), which producing anti-inflammatory cytokines and induce Th2 responses. Adipose tissue macrophages in the obese comprise a dominant cell population linked to the inflammatory process and insulin resistance. In contrast, M2 macrophages may suppress the production of proinflammatory cytokines and attenuate inflammatory processes in adipose tissue. However, few studies have provided data on the role of adipose tissue macrophages in STZ-induced diabetes. In this study, we showed that the degree of F4/80-positive macrophage infiltration increased in epididymal adipose tissue in STZ-induced diabetic mice. Our data also demonstrated that treatment with STZ increased the mRNA expression of CD86, CD206, and FIZZ1 in epididymal adipose tissue (Supplementary Figure 1).

Ang1, an angiogenic factor, also regulates adipose tissue growth and protects vascular endothelial cells from apoptotic injury. In this experiment, we evaluated the changes in vascular endothelial cell density in epididymal adipose tissue after STZ injection. The density of PECAM-1-positive vascular endothelial cells was significantly decreased after STZ injection compared to injection with control buffer. We also found that COMP-Ang1 had a protective role against the STZ-induced decrease of vascular density in epididymal adipose tissue. As COMP-Ang1 had an anti-inflammatory effect, we evaluated the changes in macrophage infiltration in epididymal adipose tissue. The degree of F4/80-positive macrophage infiltration in epididymal adipose tissue after treatment with COMP-Ang1 adenovirus was significantly decreased compared with LacZ treatment. These data suggest that COMP-Ang1 has an anti-inflammatory role in STZ-induced adipose tissue inflammation.

Su D et al [24] showed that Ang1 increased glucose-stimulated insulin release after islet transplantation. They also showed that Ang1 prevented islet cell apoptosis. Consistent with a previous report, our data also demonstrated that serum insulin level in the STZ and COMP-Ang1 treatment group was significant higher than that in STZ and LacZ treatment group. Thus, increased serum insulin level after treatment with COMP-Ang1 may have a beneficial role in glucose regulation of STZ-induced diabetic mice.

Metabolic disorders involve visceral adipose tissue more so than subcutaneous adipose tissue. It has been reported that adipokine secretion, apoptotic injury, and lipolytic/lipogenic responses differ according to the anatomical location of adipose tissue [25–27]. In this study, we examined lipid droplet size, macrophage infiltration, and vascular endothelial cells exclusively in epididymal adipose tissue. Thus, further study is required regarding changes in lipid droplet size and inflammatory processes in other adipose tissues.

The mechanism of COMP-Ang1 on the epididymal adipose tissue in this study is not clear. However, we suggest several mechanisms. The anti-inflammatory effects of COMP-Ang1 on endothelial cells in epididymal adipose tissue may have resulted in decreased macrophage infiltration and suppression of adipocyte inflammation. Increase of lipogenic effects by COMP-Ang1 treatment might be related to increase in lipid droplet size and decrease in adipokine secretion, including free fatty acid secretion. Increased serum insulin level may improve adipose tissue inflammation and metabolic-related parameters.

Diabetic nephropathy is linked to oxidative stress, and inflammation and finally leads to glomerulosclerosis [28, 29]. Direct regulation of renal inflammation may ameliorate progression of renal disease. Severity of glomerular sclerosis in the diabetic nephropathy model is usually related to diabetes duration from 1 to 4 month [30]. However, there are not many reports about anti-inflammatory effects on kidneys 6 w after STZ. In this study, we evaluated the effect on renal inflammation at 6 w after STZ injection with or without COMP-Ang1 treatment. Our data showed that administration of COMP-Ang1 decreased STZ-induced renal endothelial ICAM-1, VCAM-1 and macrophage accumulation in the kidney 6 w after STZ administration. Thus, we can suggest that COMP-Ang1 ameliorates early renal inflammation after STZ treatment.

## MATERIALS AND METHODS

### Animals

C57BL/6 mice (Charles River Korea, Seoul, Korea; 20–30 g body weight) were used in these experiments. The animal experimental protocol was reviewed and approved by the Institutional Animal Care and Use Committee of Chonbuk National University (CBU 2012-0012). Animals were anaesthetized with ketamine hydrochloride and xylazine (2.5 mg and 7.5 mg/100 g body weight, respectively) and blood samples were collected by cardiac puncture.

### Study design

Treatment protocols are summarized in Figure 1.

### Experiment 1

Male C57BL/6 mice were randomly divided into five groups. The mice in the first and second groups received citrate buffer (control buffer). The mice in the third and fourth groups were treated with STZ (STZ; Sigma-Aldrich, St. Louis, MO, 50 mg/kg body weight, pH 4.5) intraperitoneally for 5 consecutive days. Recombinant adenoviruses expressing COMP-Ang1 (1x10^9^ PFU) or LacZ (1x10^9^ PFU) were constructed as previously described [22]. Group 1 mice were injected with LacZ adenovirus in control buffer. Group 2 mice received COMP-Ang1 adenovirus in control buffer. In groups 3 and 4, mice were treated with STZ as described above, and a LacZ adenovirus (group 3) or COMP-Ang1 adenovirus (group 4) was also injected. An adenovirus expressing a soluble form of Tie2, sTie2, (1x10^9^ PFU), an inhibitor of COMP-Ang1, was injected along with STZ and COMP-Ang1 adenovirus in group 5. Blood glucose levels were measured at 0, 2, and 4 w after STZ injection. Blood samples were obtained from the tail veins of fasted mice and glucose was measured with a blood glucometer (Accu-Chek^®^ Active, Roche Diagnostics Korea, Seoul, South Korea). Mice were considered diabetic when fasting blood glucose levels were above 400 mg/dL at 2 w after STZ administration, and diabetic mice received intravenous injections of COMP-Ang1 adenovirus or LacZ adenovirus at 2 and 4 w after STZ treatment. In our previous experiments, circulating serum levels of COMP-Ang1 increased 3 days after treatment, peaked at 5 days, and declined thereafter [31]. Mice were euthanized 4 w after injection with COMP-Ang1 adenovirus and epididymal adipose tissue, kidney and blood was harvested (Figure 1).

### Experiment 2

To evaluate the preventive effects of COMP-Ang1 in STZ-induced diabetic mice, COMP-Ang1 adenovirus or LacZ adenovirus was simultaneously administered with STZ/control buffer. Diabetes was induced as in experiment 1. Male C57BL/6 mice were randomly divided into four groups as follows. Group 1 mice were injected with LacZ adenovirus in control buffer. Group 2 mice received COMP-Ang1 adenovirus in control buffer. In groups 3 and 4, mice were treated with STZ as described above in conjunction with LacZ adenovirus (group 3) or COMP-Ang1 adenovirus (group 4) injection. Blood glucose levels were measured at 0, 1, 2, 3, and 4 w after STZ injection. Mice were euthanized 4 w after injection with STZ, and epididymal adipose tissue, and blood were harvested (Figure 1).

### Immunofluorescent staining

Mice were anesthetized with a combination of ketamine and xylazine. Epididymal adipose tissues were removed, weighed, fixed with 1% paraformaldehyde in phosphate buffered saline, and whole-mounted. Immunofluorostaining was performed as previously described [21]. After blocking with 5% goat serum in 0.3% Triton X-100 in phosphate buffered saline for 1 h, the whole-mounted epididymal adipose tissue were incubated overnight at 4°C with hamster anti-PECAM-1 antibody (Chemicon International, Temecula, CA) to indicate vascular endothelial cells, and F4/80 to indicate macrophages (BD Pharmingen Bioscience, San Diego, CA). After washing in phosphate buffered saline, whole-mounted epididymal adipose tissues were incubated for 1 h at room temperature with Cy3-conjugated anti-hamster IgG secondary antibody and Cy5-conjugated anti-rat IgG secondary antibody (Jackson ImmunoResearch Laboratories, Inc., West Grove, PA). Images were taken with an LSM 510 META confocal laser scanning microscope (Carl Zeiss).

### BODIPY staining and measurement of adipocyte fat droplet diameter

Adipocytes were visualized by staining lipid droplets with the fluorescent dye, BODIPY^®^ 493/503 (Invitrogen, Carlsbad, CA) as previously described [21]. In brief, epididymal adipose tissue was incubated in a 500 μL volume of 1 μg/mL BODIPY 493/503 for 1 h at room temperature. Digital images were obtained with an LSM 510 META confocal laser scanning microscope (Carl Zeiss). The diameter of fat droplets in epididymal adipocytes was measured as previously described. The diameter of BODIPY 493/503-positive cells in each image was measured with a software program (AxioVision Rel.4.7, Carl Zeiss). We measured 300 cells in epididymal adipose tissues in each group by this method.

### Determination of free fatty acid, triglyceride, glucose, and insulin levels, and HOMA index

Free fatty acid and triglyceride levels were determined in mouse serum with a Hitachi 7180 Chemistry Analyzer (Hitachi, Japan). Fasting blood glucose was measured with an Accu-Chek^®^ Active (Roche Diagnostics Korea) and was expressed as milligrams per deciliter [32]. Mouse serum insulin levels were measured using an insulin enzyme-linked immunosorbent assay (ELISA) kit (Shibayagi Co., Ltd, Gunma, Japan). Insulin resistance was determined by the homeostasis model assessment (HOMA) method using the following equation: HOMA index = [fasting insulin (μU/mL) × fasting glucose (mmol/L)]/22.5 [33].

### Western blotting

Western blot analysis was performed as previously described [31]. Antibodies against phospho-Akt and Akt (Cell Signaling Technology, Danvers, MA), phospho-ERK and ERK (Cell Signaling Technology) were used, and signals were visualized by chemiluminescent detection according to the manufacturer’s protocol (Amersham Pharmacia Biotech, London, UK).

### Quantitative real-time PCR analysis of lipogenic and lipolytic genes

Total RNA was extracted from epididymal adipose tissue using TRI Reagent (MRC, Cincinnati, OH). After reverse transcription, quantitative real-time polymerase chain reaction (PCR) was performed using a SYBR^®^ Green PCR Master Mix (Applied Biosystems, Carlsbad, CA) on a 7900HT Fast Real-Time PCR System (Applied Biosystems) to measure lipogenic genes including *Ppar-γ, C/ebp-α*, and lipolytic genes including *Atgl* [34, 35]. The PCR program was as follows: 2 min at 50 °C, 10 min at 95 °C, then 95 °C for 15 s, and 60°C for 1 min for 40 cycles. The average threshold cycle was determined from triplicate reactions and expression levels were normalized to the housekeeping gene, *Glyceraldehyde 3-phosphate dehydrogenase (Gapdh)*, as previously described [36]. The following primers were used for PCR analysis: for *Ppar-γ*, forward primer forward primer 5′- GGAAGACCACTCGCATTCCTT-3′ and reverse primer 5′-GTAATCAGCAACCATTGGGTCA-3′; for *C/ebp-α*, reverse primer 5′-GCGGGAACGCAACAACATC-3′ and forward primer 5′- GTCACTGGTCAACTCCAGCAC-3′; and for *Atgl*, reverse primer 5′- TCCGTGGCTGTCTACTAAAGA-3′ and forward primer 5′-TGGGATATGATGACGTTCTCTCC-3′.

### Statistical analysis

Data were presented as the mean ± S.D. For parametric data, significance was determined using the Student’s *t*-test, and one- or two-way ANOVA as specified in the text, followed by Tukey’s post-hoc tests. Non-parametric data was analyzed using the Mann-Whitney U or Kruskal-Wallis tests followed by Dunn’s post-test. Statistical values of *p* < 0.05 were considered significant.

## SUPPLEMENTARY MATERIALS FIGURES


