# Supplementary material for: COMP-angiopoietin-1 mitigates changes in lipid droplet size, macrophage infiltration of adipose tissue and renal inflammation in streptozotocin-induced diabetic mice
Source: Oncotarget. 2017 Oct 23;8(55):94805–18. doi: 10.18632/oncotarget.21998 (PMC5706914; doi:10.18632/oncotarget.21998)
Supplement: Supplementary file 1 [file oncotarget-08-94805-s001.pdf]

COMP-angiopoietin-1 mitigates changes in lipid droplet size, macrophage infiltration of adipose tissue and renal inflammation in streptozotocin-induced diabetic mice

SUPPLEMENTARY MATERIALS

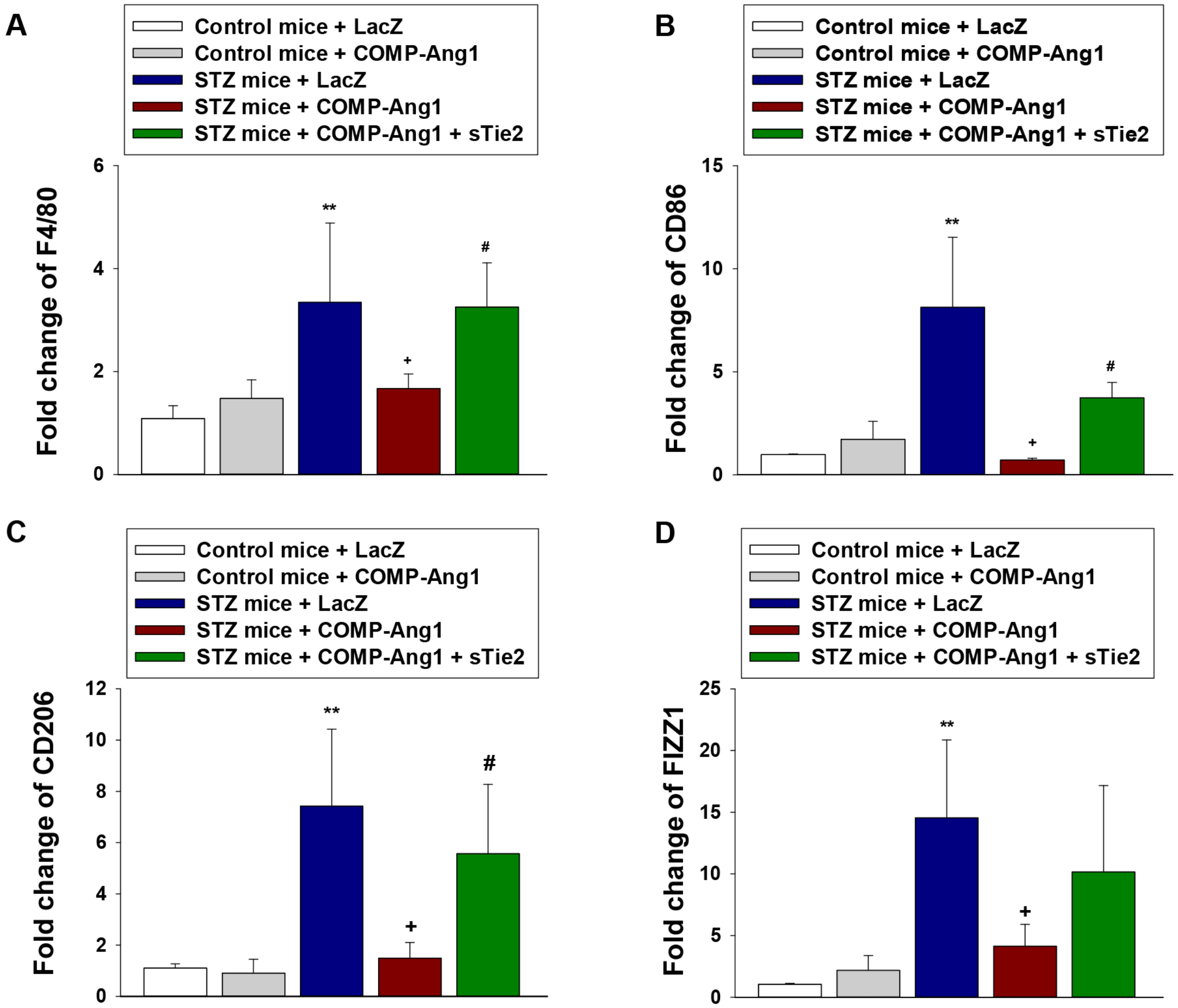

Supplementary Figure 1: (A-D) Expression levels of F4/80 (A), CD86 (B), CD206 (C) and FIZZ1 (D) mRNA in epididymal adipose tissue. mRNA expression of each gene was measured by quantitative real-time PCR (qRT-PCR). qRT-PCR was performed individually on five samples in each group. mRNA levels were measured 4 w after COMP-Ang1 adenovirus injection in control (Control mice) and STZ-induced diabetic mice (STZ mice). Data shown represent fold-change relative to Control mice+LacZ after normalization to GAPDH. $^{**}P<0.01$ vs. Control mice+LacZ; + $P<0.05$ vs. STZ mice+LacZ; # $p<0.05$ vs. STZ mice+COMP-Ang1.

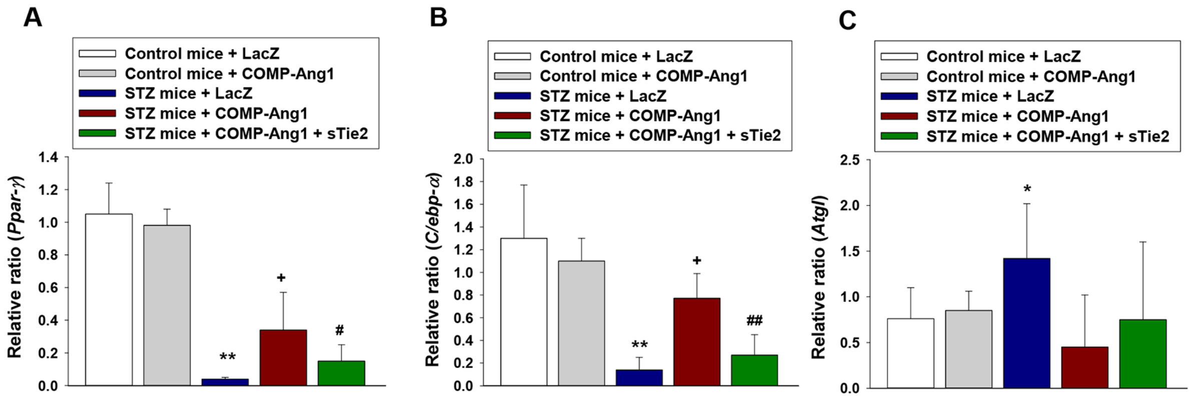

Supplementary Figure 2: Quantitative real-time PCR analyses of lypogenic genes such as *Peroxisome proliferator-activated receptor-gamma* (*Ppar-γ*, **A**), *CCAAT enhancer-binding protein-alpha* (*C/ebp-α*, **B**) and lipolytic gene such as *Adipose triglyceride lipase* (*Atgl*, **C**) in epididymal adipose tissue of mice after injection with COMP-Ang1 in control and STZ mice. Fold changes of each gene expression are relative to Control mice. Bars represent the means ± SD from 3 independent experiments. $^{*}P<0.05$ vs. Control mice+LacZ; $^{**}P<0.01$ vs. Control mice+LacZ; + $p<0.05$ vs. STZ mice+LacZ; # $p<0.05$ vs. STZ mice+COMP-Ang1; ## $p<0.01$ vs. STZ mice+COMP-Ang1.

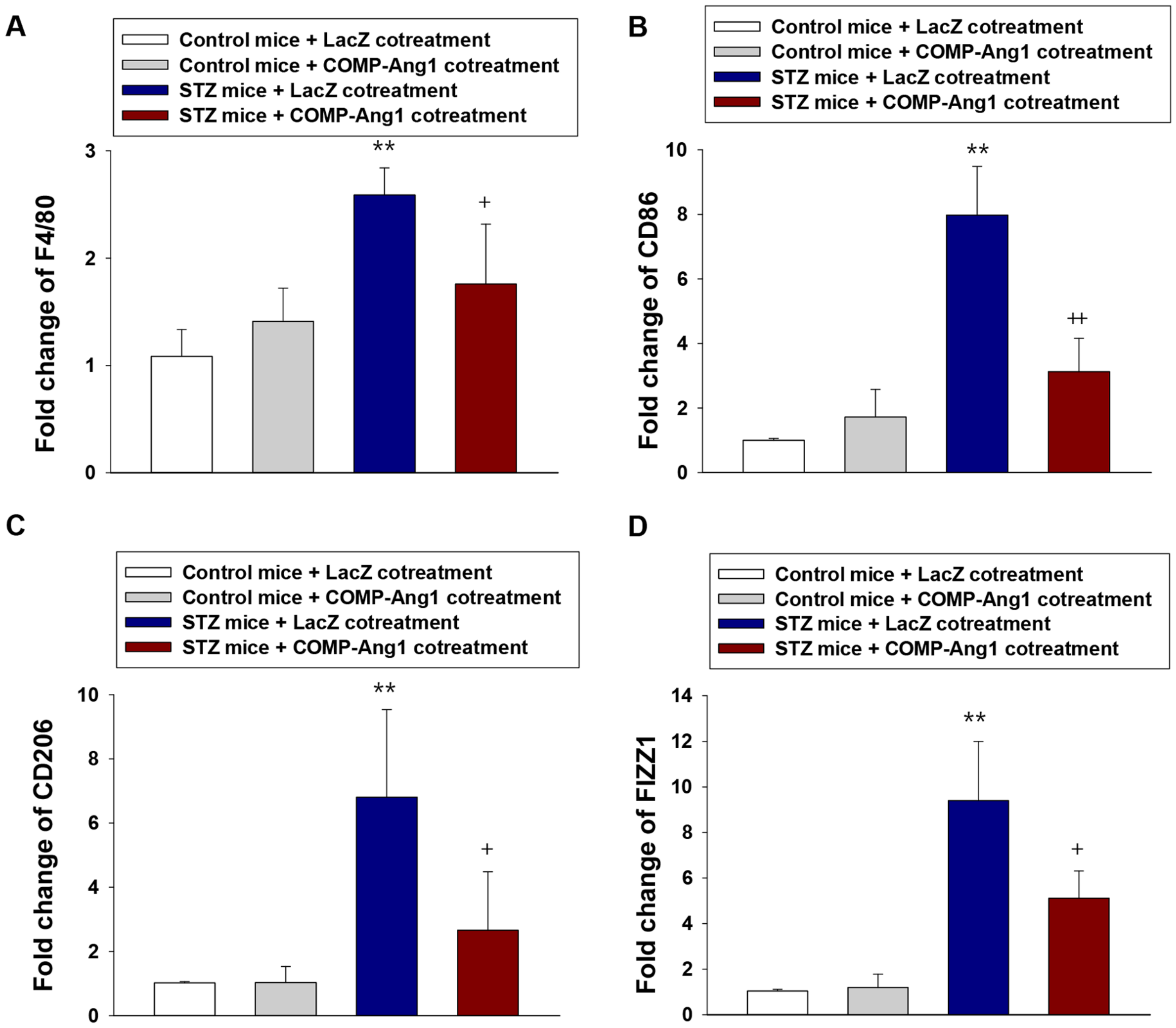

Supplementary Figure 3: (A-D) Expression levels of F4/80 (A), CD86 (B), mannose receptor (CD206, C) and FIZZ1 (D) mRNA in epididymal adipose tissue. To evaluate the preventive effect of COMP-Ang1, COMP-Ang1 adenovirus was injected simultaneously with control buffer (Control mice) or STZ (STZ mice). Adipose tissue from mice that received control buffer plus LacZ (Control mice + LacZ cotreatment), control buffer plus COMP-Ang1 adenovirus (Control mice + COMP-Ang1 cotreatment), STZ plus LacZ (STZ mice + LacZ cotreatment), or STZ plus COMP-Ang1 adenovirus (STZ mice + COMP-Ang1 cotreatment) were harvested 4 w after injection with LacZ or COMP-Ang1 adenovirus. mRNA expression of each gene was measured by qRT-PCR. qRT-PCR was performed individually on five samples in each group. Data shown represent fold-change relative to Control mice+LacZ after normalization to GAPDH. $^{**}P<0.01$ vs. Control mice+LacZ cotreatment; + $P<0.05$ vs. STZ mice+LacZ cotreatment; ++ $p<0.01$ vs. STZ mice+LacZ cotreatment.

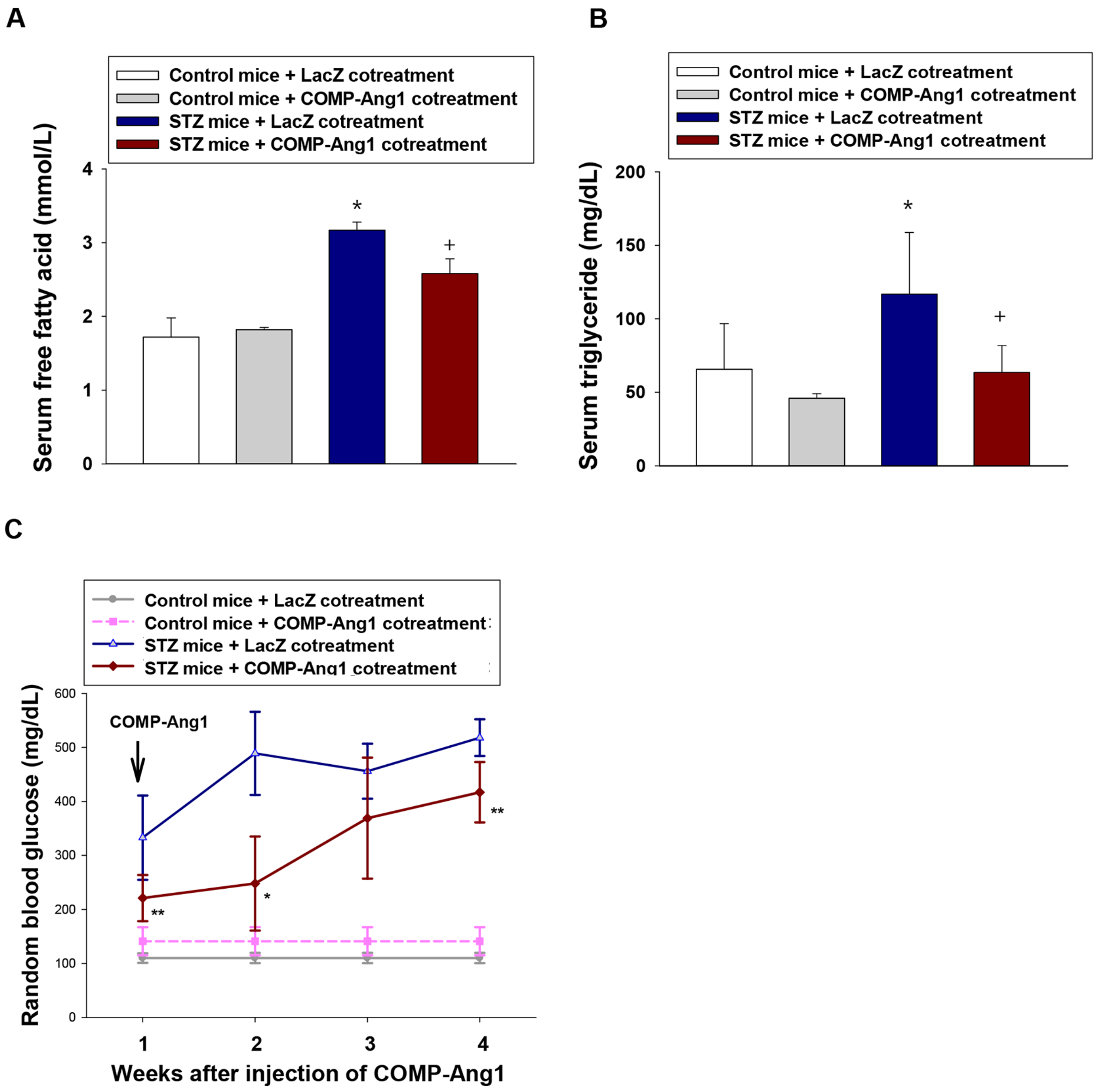

Supplementary Figure 4: (A-B) Serum levels of free fatty acids (A) and triglycerides (B). To evaluate the preventive effect of COMP-Ang1, COMP-Ang1 adenovirus was injected simultaneously with control buffer (Control mice) or STZ (STZ mice). Serum levels of free fatty acids and triglycerides were measured 4 w after injection with LacZ or COMP-Ang1 adenovirus. The values are the means ± SD for four animals in each group. **(C)** Changes in fasting blood glucose 4 w after injection with COMP-Ang1 in Control and STZ mice. Data are means ± SD for four animals in each group. $^{*}P$<0.05 vs. Control mice+LacZ cotreatment; $^{**}P$<0.01 vs. Control mice+LacZ cotreatment; + *P*<0.05 vs. STZ mice+LacZ cotreatment; ++*P*<0.01 vs. STZ mice+LacZ cotreatment.
